# Commentary: Pathophysiological Role of Histamine H4 Receptor in Cancer: Therapeutic Implications

**DOI:** 10.3389/fphar.2020.00874

**Published:** 2020-06-05

**Authors:** Abdelhakim Salem, Tuula Salo

**Affiliations:** ^1^Department of Oral and Maxillofacial Diseases, Clinicum, University of Helsinki, Helsinki, Finland; ^2^Translational Immunology Research Program (TRIMM), Research Program Unit (RPU), University of Helsinki, Helsinki, Finland; ^3^Cancer and Translational Medicine Research Unit, University of Oulu, Oulu, Finland; ^4^Department of Oral Pathology, Helsinki University Hospital (HUS), Helsinki, Finland

**Keywords:** Histamine, Histamine H4 receptor, Oral Cancer (OC), Oral tongue cancer, head and neck cancer

The past two decades have witnessed a substantial increase in our understanding of histamine interaction and functions. The main factor propelling pharmacological interests in histamine research is its pleiotropic pathophysiological actions, which have led to the development and marketing of many successful medications for immune and gastrointestinal disorders (Tiligada and Ennis, 2020). A large body of research evidence has recently revealed a crucial role of histamine in cancer-related processes such as cell cycle and proliferation, apoptosis and invasion (Massari et al., 2020). Moreover, the discovery of the latest G protein-coupled receptor of histamine, histamine H4 receptor (H4R), and its potent immunomodulatory effect, has opened up new perspectives in cancer research (Massari et al., 2020). Besides its distribution on immune cells, H4R was also differentially expressed in a wide variety of cancers, cancer models, and tumor cell lines. In this regard, we read with great interest the elegant work of Nicoud et al., which comprehensively reviewed the recent research on the pathophysiological role of H4R in cancer (Nicoud et al., 2019). The authors summarized the current knowledge on H4R pharmacology, clinical applications, and differential expression in many *in vitro* and *in vivo* experimental models of cancer as well as in cancer patient cohorts. Importantly, authors concluded that numerous preclinical data indicated with no doubt the participation of H4R in tumor progression and signify the potential of exploiting this receptor for cancer treatment. In this respect, we would like to briefly highlight the involvement of H4R in oral (or mobile) tongue squamous cell carcinoma (OTSCC), which however was not covered in this review.

OTSCC is the most common malignancy in the oral cavity, which mainly arises in the lateral borders of tongue and shows a swift metastasis to the regional lymph nodes. The prognosis of OTSCC, despite the improvement in cancer therapy, remains low (the 5-year survival rate is about 50%) without major improvements in survival within the past decades (Chen et al., 2018). Indeed, early diagnosis and effective treatments, with less off-target effects, are imperative to improve the survival rates of OTSCC patients (Chen et al., 2018). Thus, there is an urgent need to identify, test, and develop new effective therapeutic targets for OTSCC. Histamine and H4R showed differential immunoexpression in the tumor microenvironment and adjacent non-neoplastic tissue of many cancer types (Massari et al., 2020). In this sense, we demonstrated the functional expression of H4R in cultured human oral keratinocytes (HOKs) and also the presence of H4R in OTSCC specimens (Salem et al., 2015; Salem et al., 2017a). However, the intensity of H4R staining in normal oral epithelium was significantly higher than in OTSCC tissues.

Human mast cells represent the main precursor of histamine and constitutively express H4R, which participates in regulating autocrine and paracrine histamine-mediated actions (Lippert et al., 2004). Upon stimulation, mast cells produce histamine in high “burst-released” quantities that can induce pathophysiological effects mainly *via* the classical histamine receptor subtypes (H1R and H2R). On the other hand, other cell types, such as T lymphocytes and epithelial cells, can synthesize and release nearly 1,000-fold less amount of histamine, which mediates physiological effects mainly through the high-affinity receptor subtypes (H3R and H4R) (Konttinen et al., 2013). Therefore, our group has coined a new term to differentiate between these two cell categories based on their capacity to synthesize and produce histamine (Konttinen et al., 2013). In this regard, cells that are able to produce and store large (often micromolar) concentrations of histamine in secretory granules (e.g. mast cells, basophils, and enterochromaffin-like cells) were termed “professional histamine producing cells.” Alternatively, cells that exhibit no capacity to store the newly formed histamine, but they transport it passively to the extracellular milieu in a concentration gradient manner, were termed “non-professional histamine producing cells” (Salem et al., 2017b).

Interestingly, cultured HOKs can release low amounts of histamine to the extracellular environment, which is able to sensitize the high-affinity H4R in autocrine and paracrine modes (Salem et al., 2017b). Thus, HOKs were considered as non-professional histamine-producing cells. In OTSCC patients, the H4R immunoreactivity was inversely correlated with the histopathological grade of the specimen and with the numbers of the professional histamine producing mast cells. Furthermore, the expression of H4R at both receptor and gene levels was reduced in two different OTSCC cell lines compared with the normal HOKs (Salem et al., 2017a). OTSCC can arise from dysplastic and potentially malignant lesions, such as oral lichen planus, which also showed lower expression of H4R and increased numbers of mast cells compared with non-dysplastic tissue (Salem et al., 2017a) (**Figure 1**). More importantly, supernatants collected from cultured human mast cells showed regulatory effects on oral oncogenes in HOKs (e.g., epidermal growth factor receptor), while high concentration of mast cell-derived histamine downregulated H4R gene in the same cell lines (Salem et al., 2015; Salem et al., 2017a). The local concentrations of histamine in tumor tissue can influence the specificity of histamine responses (Konttinen et al., 2013). In this context and based on the implications stemming from the aforementioned classification of cells based on their histamine production capacity, we encourage the authors and field researchers to consider the role of tumor-associated mast cells when assessing H4R expression in different stages of tumorigenesis.

**Figure 1:**
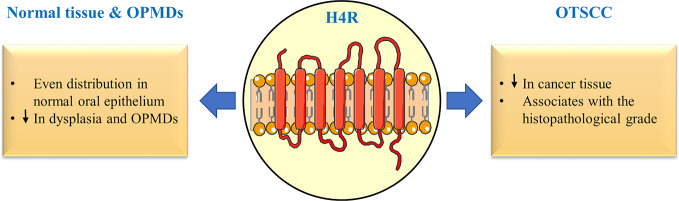
Differential expression of histamine H4 receptor (H4R) in oral potentially malignant disorders (OPMDs) and oral tongue squamous cell carcinoma (OTSCC).

The role of H4R in oral tumorigenesis is not yet fully understood. However, according to the currently available data, H4R seems to impart antitumorigenic properties and plays a crucial role in the maintenance of normal epithelium. Thus, H4R agonists could offer promising therapeutic target in the treatment of solid tumors (Martinel Lamas et al., 2013). However, we acknowledge the limitations of our studies including a limited sample size and lack of animal model experiments to validate the *in vitro* findings, and thus further studies are warranted. In addition, the selectivity of some H4R antibodies poses an additional limitation in H4R-research, as there are considerable structural similarities between H4R and H3R. Finally, we perfectly agree with the authors that several challenges remain to be addressed in H4R-based translational research. Such limitations include, but are not limited to, ligand selectivity, experimental models' heterogeneity, and the undetermined specificity of many commercially available antibodies (Nicoud et al., 2019). Nevertheless, the ongoing extensive investment in H4R research indicates that exploiting this receptor in cancer therapy could be feasible in the near future.

## Author Contributions

AS drafted the manuscript. TS reviewed and edited the manuscript.

## Funding

This work was supported by Emil Aaltonen Foundation (Emil Aaltosen Säätiö); The Maud Kuistila Memorial Foundation; The Jane and Aatos Erkko Foundation; and Helsinki University Central Hospital (HUS) Research Funds.

## Conflict of Interest

The authors declare that the research was conducted in the absence of any commercial or financial relationships that could be construed as a potential conflict of interest.
